# Ten lessons learnt: scaling and transitioning one of the largest mobile health communication programmes in the world to a national government

**DOI:** 10.1136/bmjgh-2021-005341

**Published:** 2021-07-22

**Authors:** Sara Chamberlain, Priyanka Dutt, Anna Godfrey, Radharani Mitra, Amnesty Elizabeth LeFevre, Kerry Scott, Jai Mendiratta, Vinod Chauhan, Salil Arora

**Affiliations:** 1 Asia, BBC Media Action, New Delhi, India; 2 Asia, BBC Media Action, London, UK; 3 International Health, Johns Hopkins University Bloomberg School of Public Health, Baltimore, Maryland, USA; 4 Independent Researcher, Bangalore, Karnataka, India; 5 Digital, BBC Media Action, New Delhi, India

**Keywords:** health education and promotion, child health, health systems, maternal health, public health

## Abstract

There has been exponential growth in the numbers of ‘digital development’ programmes seeking to leverage technology to solve systemic challenges. However, despite promising results and a shift from pilots to scale-ups, many have failed to realise their full potential. This paper reflects on lessons learnt from scaling and transitioning one of the largest mobile health programmes in the world to the Indian government. The complementary suite of services was designed by BBC Media Action to strengthen families’ reproductive, maternal, neonatal and child health behaviours. Mobile Academy was a training course to refresh frontline health workers’ (FLHWs) knowledge and improve their interpersonal communication skills. Mobile *Kunji* was a job aid to support FLHWs’ interactions with families. *Kilkari* delivered weekly audio information to families’ phones to reinforce FLHWs’ counselling. As of April 2019, when Mobile Academy and *Kilkari* were transitioned to the government, 206 000 FLHWs had graduated and *Kilkari* had reached 10 million subscribers. Lessons learnt include the following: (1) private sector business models are challenging in low-resource settings; (2) you may pilot ‘apples’ but scale ‘oranges’; (3) trade-offs are required between ideal solution design and affordability; (4) programme components should be reassessed before scaling; (5) operational viability at scale is a prerequisite for sustainability; (6) consider the true cost of open-source software; (7) taking informed consent in low-resource settings is challenging; (8) big data offer promise, but social norms and SIM change constrain use; (9) successful government engagements require significant capacity; (10) define governance structures and roadmaps up front.

**Summary box**

- We present lessons learnt from scaling and transitioning one of the largest mobile health communications programmes in the world to the government of India, which represent a rare example of how to achieve digital sustainability at scale.
- The hard realities of delivering public health services at scale with constrained resources may require a much more pragmatic approach than many digital pilots employed.
- Solutions should be designed based on the limits imposed by sustainability, which may require compromises in the intensity, frequency and targeting of an intervention—or ultimately risk unaffordability.
- Patience and sustained investment—particularly in monitoring, learning and evaluation—are required to amplify the impact of digital solutions at scale.

## Introduction

Over the past decade, there has been exponential growth in the number of ‘digital development’ programmes seeking to leverage technology to solve systemic challenges in almost every development domain, including healthcare.^1^ However, although investors have made a notable shift in recent years from funding pilots to scale-ups, many digital interventions still struggle to achieve scale and sustainability.^2^ Much has been written about the causes of ‘pilotitis’, a term used to describe the predominance of small digital development interventions that never scale and die the day the original funding runs out.^3^ The digital development community has also invested significant effort in articulating the cure to pilotitis—the principles of effective digital development.^4^ Nonetheless, key principals continue to be overlooked. This is perhaps because some remain largely theoretical in a context where few real-world examples of digital development interventions achieving not just scale, but sustainability, exist.

In India, there are notable examples of digital health solutions successfully scaling across geographies to change health practices and generate demand for supply side services from the last decade. For example, a suite of complementary mobile health (mHealth) services designed between 2011 and 2013 by BBC Media Action in the state of Bihar have had an impact on a range of health outcomes at scale.^5^ These are: Mobile Academy, an Interactive Voice Response (IVR)-based training course to refresh frontline health workers’ (FLHWs) knowledge and improve their interpersonal communication skills. Mobile *Kunji*, an IVR and print-based job aid to support FLHWs’ interactions with families, and *Kilkari* which delivers stage-based, time-sensitive, weekly audio information directly to families’ phones to reinforce FLHWs’ counselling. As of April 2019, when Mobile Academy and *Kilkari* were transitioned to the national government, 206 000 FLHWs had graduated and *Kilkari* had reached 10 million subscribers. Although Mobile *Kunji* was used by 144,000 registered FLHWs for 7 years in Bihar, scaled to the states of Odisha and Uttar Pradesh, and had a significant impact on a range of health outcomes, it was not adopted at the national level.^6^

This paper reflects on the lessons learnt from designing, scaling and transitioning Mobile Academy and *Kilkari* to the national government in India, and why Mobile *Kunji* was not scaled. We first describe (1) why a user-paid business model failed to cover all programmatic costs in Bihar. We then discuss our pivot to a government-paid business model, and the following key learning: (2) you may pilot ‘apples’ but have to scale ‘oranges’; (3) trade-offs are required between ideal solution design and affordability; (4) programme components should be reassessed before scaling; (5) operational viability is a prerequisite for sustainability; (6) consider the true cost of open-source software; (7) taking informed consent in low-resource settings is challenging; (8) big data offer promise, but social norms and SIM change constrain use; (9) successful government engagements require significant capacity; (10) define governance structures and roadmaps up front.

## User-paid business models are challenging in low-resource settings

mHealth projects rarely test the viability of sustainability strategies, including business models, at the pilot stage. Instead, they develop roadmaps for sustainability, which are often only implemented towards the end of a grant. At that point, it can be difficult to pivot if sustainability plans fail. In 2012, BBC Media Action began road-testing a user-paid business model, which involved revenue share agreements with six mobile network operators (MNOs) in Bihar, while it still had 3.5 years of donor funding left. It adopted this approach to test if it was feasible to ensure the sustainability of the services without funding from donors or the government.

In 2011, formative research was conducted to identify willingness to pay for the suite of mHealth services. It identified that, although FLHWs were not willing to pay for a job aid they used every day (Mobile *Kunji*), they were willing to pay approximately US$1.50 for the entire Mobile Academy course, billed on a ‘pay as you go’ basis at US$0.01 per min. It also found that mothers did not make financial decisions related to the phone, and fathers, who spent an average of US$1 a month on talk time, were only willing to pay approximately US$0.0125 per week for *Kilkari* for 18 months. They were not willing or able to pay the US$1 that it would cost to buy the entire service up front, because on average, mobile subscribers in Bihar in 2011 only spent $1 on phone credit per month.

Most of the revenue generated by calls to Mobile Academy and from *Kilkari* went to the MNOs to cover their operating costs, which included the cost of maintaining their network infrastructure. Under the typical value-added services revenue share model in India in 2011, MNOs took 80%–90% of the revenue—leaving only 10%–20% to cover ongoing costs, including marketing. Although 50 000 FLHWs paid to complete Mobile Academy from their own pockets, and more than 168 969 families subscribed to receive reproductive, maternal, newborn and child health (RMNCH) advice from Kilkari at a cost of US$0.01 per week, this model did not generate enough revenue to cover the high cost of face-to-face marketing, in addition to operating costs.

Face-to-face marketing proved necessary to reach low literate pregnant and postpartum women and their families in low-income, rural communities because digital marketing and marketing through the MNOs’ top-up shops mainly reached young men who either were not married or did not see the value in paying for preventative RMNCH information. Due to the high cost of acquiring subscribers, and the low price point that poor families could afford, this business model failed to cover all costs. But at least it ‘failed fast’, giving the team time to pivot to a government-paid business model. Under this new model, the government covered all call costs, making the service free to subscribers. Thanks to economies of scale, the government was also able to negotiate less expensive call rates with a single MNO for the suite of mobile health services. To identify and subscribe beneficiaries at scale, the programme integrated *Kilkari* with the government databases that track pregnancies and births in India. This enabled the automated subscription of millions of new and expecting mothers with no investment in marketing.

Elsewhere globally, maternal messaging programmes in South Africa^7^ and Ghana^8^ have established vertical systems for enrolment into messaging programmes subscribing beneficiaries through face-to-face interactions with healthcare providers in the public sector. While face-to-face encounters provided an opportunity for providers to describe the programme to prospective beneficiaries, answer queries and capture informed consent, it too has cost implications for promoting the programme’s rollout in health facilities and training providers on registration procedures. In addition, implementation of face-to-face registration may have opportunity costs with regard to provider and beneficiary time, extending beneficiary time in the clinics and shifting provider time away from clinic care to enrolment and registration during the clinical encounter. The *Kilkari* programme’s ability to automate subscription based on data drawn from government-tracking registries has meant that large numbers of women at a population level can enter the programme with no additional burden to existing health systems or the clinical care received by women and children.

## You may pilot ‘apples’ but have to scale ‘oranges’

Large complex interventions that include integrated theories of change need to be reassessed if only some of these interventions are scaled. In Bihar, an overarching theory of change was developed for a layered communications programme. The resulting programme combined mass media, community mobilisation, interpersonal communication and mobile-based solutions to extend vital health information and advice to rural populations.^5^ The complementary suite of three mHealth services were nested under this broader programmatic theory of change and were designed under a single digital theory of change. They aimed to (1) improve FLHWs knowledge of preventative RMNCH behaviours and strengthen their interpersonal communications skills, (2) equip FLHWs with a high-quality job aid to support and standardise their interactions with families, and (3) provide new and expecting mothers and their families directly with audio information to reinforce messages communicated by FLHWs.

When the national government scaled the mHealth services, it did so without the wider interventions including Mobile *Kunji,* which was not scaled because it was thought that smartphone job aids would make the IVR and print-based tools obsolete. Thus arguably *Kilkari*, which was designed to reinforce the information communicated by FLHWs and urges women to ask FLHWs questions about the content, may be even more effective in states and districts where FLHWs are also equipped with job aids to improve their communication skills, or where the environment is more supportive of behaviour change.^5^

Additionally, when *Kilkari* was adopted by the national government, new versions of the content had to be created to engage millions of new and expecting mothers and their families in multiple states, and to address national and state government priorities. User testing in Bihar had revealed that to improve comprehension and recall, information should be limited to one, simple, doable action per *Kilkari* call. However, when going to scale that was not always feasible and it was necessary to communicate several simple doable actions in each call.

## Trade-offs are required between ideal solution design and affordability

The hard realities of delivering public health services at scale with constrained resources may require a much more pragmatic approach than many pilots employ. In a donor-funded pilot project, it is possible to design, test and develop an ‘ideal’ maternal messaging programme—particularly if the donor covers the cost of calls or SMS messages. For example, for *Kilkari,* trade-offs were required in three areas: (1) number of calls; (2) degree of language localisation; (3) times of day when calls were made to subscribers.

In pilots, hyperlocalised content can be created for a small sample of women. Skilled project staff can support women who own phones in registering for the service. Accurate mobile numbers and the estimated due date of a child can be captured using software, devices and connectivity procured by the project. Data can flow into an independent, standalone database designed specifically for the service. Calls can be delivered at times of day that women find convenient. And finally, the project can send as many messages as it likes, so long as a donor is willing to pay. In the real world, however, ‘ideal’ programme design is rarely possible.

When the Indian government decided to scale Mobile Academy and *Kilkari* nationally, several important compromises to the service design were required to accommodate its budget and procurement policies, and to overcome the challenges involved in trying to reach low-income illiterate women. It is critical that digital development solutions are designed based on the limits imposed by sustainability. This approach requires compromises. Otherwise, there is a significant risk that proposed digital solutions will ultimately be unaffordable.

### Number of lessons

The ongoing operating costs of Mobile Academy and *Kilkari* were driven by the number of lessons and calls, respectively. During the pilot in Bihar, in-depth interviews indicated that families were only willing to pay for one *Kilkari* call per week, and FLHWs were only will to pay for a limited number of Mobile Academy lessons. Similarly, when the national government decided to adopt the services, it understandably expressed reservations about increasing the frequency of *Kilkari* calls from one to two per week because this would have doubled call costs and considerably increased the cost of the project’s technical infrastructure, which was already significant. Securing approval for *Kilkari* call costs at scale was already challenging at this early stage in the government’s adoption of the service. It was therefore agreed that families would receive just one *Kilkari* call per week to limit costs to an acceptable level.

### Language localisation

The Cenus of India 2011 recorded 19,569 mother tongues, including 121 languages.^9^ Even in the so-called ‘Hindi-speaking belt’, people speak many other languages and dialects. User testing in five Hindi-speaking states revealed that rural women had a much poorer comprehension of Hindi than men, largely because they lived in media-dark homes and rarely left the confines of their compounds (Chamberlain *et al*, 2020). Rural men, in contrast, spent time in public spaces talking to a wider variety of people with different accents and dialects, and had greater exposure to shared radios and televisions. Efforts to meet the linguistic needs of all women in the Hindi-speaking belt were not financially viable at scale. As a result, a lowest common denominator Hindi that could be understood by the largest number of women was created.

### Timing of calls

When *Kilkari* was only live in eight districts in Bihar, new and expecting mothers were called when they said they wanted to receive calls—that is, early in the morning, after lunch and in the early evening. However, when the government decided to scale up the service nationally, this approach was not considered financially viable. Instead, it was decided that *Kilkari* should make calls throughout the day—from 08:00 to 20:30—to maximise the utilisation of infrastructure and connectivity. Calling subscribers just three times a day would have involved making millions of calls in a much shorter window of time, which would have required significantly more infrastructure and connectivity to handle the load during those hours. Furthermore, this infrastructure and connectivity would have sat idle for much of the day when calls were not being made. Therefore, *Kilkari* was redesigned so that its infrastructure and connectivity were continuously used. This is a more cost-efficient approach, but it may result in calls being made at times of day when women are unable to answer phones.^10^

## Programme components should be reassessed before scaling

A digital development solution may need to be entirely redesigned before it can be scaled. Integration with government systems and databases, for instance, may require significant changes to technical architecture. The overall solution may also need to be simplified, as too many moving parts are challenging for governments to procure and sustain.

Before the scale up begins, it is important to reassess existing technical partners’ strengths and weaknesses. Do they have the skills, experience and—critically—staff capacity required for a national scale-up? Do they have senior engineers in-country to not only support but further develop the software? Are their licensing models cost-effective at national scale and do they allow for the transfer of ownership to the government at no additional cost?

## Consider the true cost of open-source software

One of the mantras of the digital development community is the value of open-source technologies as ‘global goods’.^4^ Based on the experience of this programme, it may prove not just more expensive to scale, enhance and support open-source software than to purchase unlimited capacity, in perpetuity licences for proprietary software applications, but also more challenging to transition open-source software to the government.

A key source of revenue for proprietary software companies is the sale of annual maintenance contracts. Their business models depend on providing cost-effective, reliable support in the countries where their software is sold. But when it comes to open-source software, sometimes the only people capable of maintaining and further developing the software are those who built it. And there may be very few of those people. Moreover, it can be just as challenging for a government to adopt open-source software as proprietary software. If there is only one company capable of (re)developing and maintaining an open-source solution, government procurement policies may prevent it from contracting this company, because at least three valid bids from different providers may be required.

Decisions to use open-source software at scale should be based not just on the functionality and performance of the software, but also on the size and sustainability of its user community, the availability of expert technical support in-country, the clarity of code and code labelling, and the quality of software documentation and user manuals. Propriety software can be just as—if not more—cost-effective than open-source software if the correct licences are negotiated. This programme negotiated one-off payments for unlimited capacity, in perpetuity software licences for Mobile Academy and *Kilkari* because they were cost-effective at scale. Critically, the programme ensured that these licenses could be transferred to the Indian government at no additional cost.

## Operational viability at scale is a prerequisite for sustainability

Mobile Academy and *Kilkari* were compelling candidates for government adoption not just because these services offered solutions to known challenges, but because they had demonstrated sustained demand from target populations, and could be set up and managed centrally with little face-to face training and were relatively straightforward and inexpensive to sustain. Mobile *Kunji*, which had also demonstrated sustained adoption by FLHWs at scale in three Indian states, and a significant impact on multiple health outcomes,^6^ was more challenging than Mobile Academy and *Kilkari* to adopt because it would have involved the procurement and distribution of a physical deck of printed cards (to accompany the IVR component of the job aid) to a million FLHWs across the nation; at least a day of face-to-face training and significant IVR call costs.

Sources at the Ministry of Health and Family Welfare (MoHFW) say the government is routinely bombarded by pitches for complex digital solutions that involve many moving parts. But few digital development pilots are designed with government procurement policies, interoperability and operational feasibility at scale in mind. Furthermore, sustainability strategies tend to focus on capital investment rather than ongoing running costs and logistics. Future mHealth projects will require more emphasis on developing strategies for effectively responding to government procurement and distribution challenges, and on costing viable operational models at scale.

## Taking informed consent in low-resource settings is challenging

When *Kilkari* was first tested in eight districts in the state of Bihar in 2013, users dialled a toll-free number to subscribe to the service. In keeping with the regulations of the Telecom Regulatory Authority of India (TRAI), they had to confirm their subscription twice by pressing numbers on their mobile keypads. This requirement excluded many women who could not make outbound calls or navigate IVR prompts. The project then experimented with using a call centre to take informed consent from subscribers. But this was equally challenging, as rural women—who had never heard of the internet or data—struggled to understand the concept of ‘informed consent’. When the MoHFW decided to scale *Kilkari* nationally, making it free to all pregnant and postpartum women, it received an exemption from TRAI consent guidelines. It was thought that the benefits of rapidly disseminating free health information outweighed concerns around data protection. As a precaution, however, users were given the ability to unsubscribe at the end of every call.

The National Health Mission in each state planned to train FLHWs to inform pregnant women and mothers of babies about *Kilkari* and to equip them to answer questions about the service. But because this sensitisation did not happen in some locations, families were caught off-guard when they suddenly started receiving *Kilkari* calls. Although many were pleasantly surprised by the arrival of *Kilkari* in their lives, others immediately unsubscribed from the service or disconnected the calls, thinking they were spam. As concerns about data protection and privacy grow among the citizens of developing countries, governments and non-governmental organisations will need to identify how to take informed consent to capture personal and sensitive data in a cost-effective way, without excluding potential beneficiaries. The pathway to achieving this has yet to be defined but may benefit from learning elsewhere. Efforts are underway in South Africa as part of the MomConnect national maternal messaging programme to enhance data capture consent language and processes. Cognitive interviews that are being conducted with pregnant women and providers are anticipated to yield revised informed consent language, which can be used to enable data use for a range of purposes, including sending health information messages, conducting health services research and recontacting beneficiaries.

## Big data offer promise, but social norms and SIM change constrain use

India’s nationwide effort to register pregnancies, births and the provision of RMNCH services, and to maintain an up-to-date FLHW database is a Herculean task. In addition to exceedingly high rates of SIM change among rural populations (44% see LeFevre *et al*, 2021^11^), the gender gap in both mobile phone access and digital literacy is a significant barrier. In a context where only 14% of adult women pan India own a smartphone^10^ and only 56% have access to the household phone in states such as Madhya Pradesh,^12^ the mobile numbers associated with women in government databases are likely to belong to male family members. Digital development solutions that rely on accurate mobile numbers for women should proceed with caution, designing content that not just speaks to mothers, but also fathers, because they may be equal if not dominant participants in the mHealth programme.

## Significant government engagement requires significant capacity

When the programme first designed the suite of mHealth services in the state of Bihar, it worked closely with the state government on strategy, content and training, but procured and delivered the services independently. This changed when the states of Odisha and Uttar Pradesh decided to adopt Mobile Academy and Mobile *Kunji*, and then again when the MoHFW decided to scale Mobile Academy and *Kilkari* nationally. At these points, the team shifted to supporting first state governments and then later the national government in directly procuring the elements of each mHealth service. This proved resource-intensive and time-consuming. The programme needed to dedicate considerable staff resources, for much longer, to support government departments in launching the services and rolling them out. For example, it took 6 months of sustained engagement to agree the scope of the scale-up and to receive approval to proceed with the implementation. It is critical that digital implementers—and their investors—are realistic about the timelines and resources required for government adoptions and scale-ups.

## Define governance structures and roadmaps up front

Key learning from a decade of designing and scaling digital solutions in partnership with state and national government indicates the critical importance of: (1) identifying how the proposed intervention can support priority government programmes; (2) working with the strongest possible government sponsor from day 1, (3) agreeing roles and responsibilities for all stakeholders up front, (4) agreeing a shared strategy for co-management, including the establishment of a programme management unit and regular joint project review meetings. Furthermore, (5) buy-in is enhanced if the government has a financial stake in the scale-up at the outset, and (6) agreeing a clear roadmap at the outset for the transition of ownership to government is key, but be prepared to adapt to unexpected challenges and opportunities.

## Conclusion

Mobile Academy and *Kilkari* represent two of the few examples, globally, of how to achieve digital sustainability at scale. Implementation lessons learnt from this journey are a reality check. They underline the criticality of reassessing every aspect of an ideal pilot programme before scale-up, and the recognition that compromises may need to be made in service design. Making a digital programme affordable is likely to involve sacrifices, which may reduce the intensity, frequency and precise tailoring or targeting of an intervention. Patience and sustained investment—particularly in learning and evaluation—are required to optimise digital solutions at scale and amplify their impact. Hasty transitions may result in lost opportunities. Furthermore, scale-ups and transitions are not controlled scientific experiments. The expectation that a pilot project will be replicable at significant scale is wishful thinking. Public sector procurement will cause delays, funding streams will no longer be in sync and different project components may not happen on time. Planning for these challenges and having the resilience to adapt to the unforeseeable should be regarded as a sign of mHealth maturity.

## Data Availability

Data are available upon request.
